# Breaking the silence: the role of sexual communication on quality of life in women with cervical cancer

**DOI:** 10.1007/s00520-024-08713-x

**Published:** 2024-07-10

**Authors:** Magdalena Liberacka-Dwojak, Monika Wiłkość-Dębczyńska, Radosław Perkowski

**Affiliations:** 1 Department of Psychology, Kazimierz Wielki Unviersity, Bydgoszcz, Poland; 2 Department of Geriatrics, Collegium Medicum in Bydgoszcz, Nicolaus Copernicus University in Toruń, Bydgoszcz, Poland

**Keywords:** Cervical cancer, Quality of life, Sexual communication

## Abstract

**Introduction:**

Cervical cancer (CC) and its treatments impact various dimensions of quality of life (QoL), including physical, psychological, and sexual functioning. Sexual health, a crucial QoL aspect, is often compromised, highlighting the necessity for open sexual communication.

**Materials and methods:**

This study involved 60 women diagnosed with stage IIb-IIIa CC. The 36-Item Short Form Survey (SF-36) was used to measure physical and psychological components of QoL, the Female Sexual Function Index-6 (FSFI-6) to assess its sexual component, and the Sexual Communication Self-Efficacy Scale (SCSES) to diagnose communication confidence. Self-administered questions gauged patient-provider sexual communication. The results include quotes from participants, providing additional insight.

**Results:**

Sexual communication self-efficacy and patient-provider communication correlated significantly with QoL components. Only 23.33% discussed sexual issues with their doctors. Participants’ experiences highlighted the impact of cancer on sexuality and the need for tailored support.

**Discussion:**

Post-diagnosis psychosexual changes emphasize the importance of communication in renegotiating sexual identity and needs. Effective communication is associated with improved QoL, highlighting the role of healthcare professionals in addressing psychosexual issues. Integrating PLISSIT and BETTER models provides a comprehensive approach to sexual communication in the cancer context.

**Conclusion:**

The study demonstrates the importance of sexual communication self-efficacy in QoL of CC patients and highlights the need for healthcare professionals to include sexual communication education in cancer care.

## Introduction

Cervical cancer (CC) and its treatment are known to have negative effects on both physical and mental well-being, including self-esteem, body image, and social functioning, which can lead to health problems [1–3]. Despite improvements in diagnosis and treatment increasing life expectancy, addressing quality of life (QoL) issues is critical [4]. Sexual health is an essential aspect of QoL [2], but while the impact of cancer on QoL is well-understood, its influence on sexuality is still an overlooked topic. Gynecological cancers often result in sexual dysfunctions due to the treatment, the cancer itself, and the psychological responses to the diagnosis [5].

As awareness of the side effects of cancer treatment is increasingly growing [6], there is a constant need to investigate potential factors that can enhance QoL. Open sexual communication can be considered a crucial element in preserving and enhancing sexual health, as well as fostering satisfaction in relationships and psychological well-being. This communication allows for positive adaptation to cancer complications [2, 7].

Recent research indicates that a significant majority of patients expect to discuss with their oncology professionals sexual issues. Unfortunately, only a small percentage of patients actually receive the necessary information from their healthcare providers [5, 8]. Furthermore, recent studies suggest that merely one-third of cervical cancer patients, who require psychosexual support, take the initiative to discuss these matters with a professional [9]. Excluding this aspect from the medical approach leaves patients without both essential support and information regarding treatment options. This omission can potentially intensify emotional and sexual difficulties, as well as contribute to relationship concerns. Recent recommendations emphasize the value of incorporating sexual health into the multidisciplinary approach for oncology patients [10].

Self-efficacy in sexual communication is crucial for open discussions with partners, children, and healthcare providers. This involves a combination of sexual self-disclosure, assertiveness, sexual values, and the frequency and quality of communication. Low self-efficacy can result in perceived barriers and difficulties in addressing sexual topics in both partner-partner relationships and the patient-provider context. The roots of this issue are diverse, encompassing factors such as shame, undervaluing the importance of sexual discussions, feelings of insecurity, or fear of causing harm to one’s partner [11–13].

Effective sexual communication is thought to improve QoL by receiving support from both partners and healthcare providers [14]. This study aimed to identify the role of sexual communication in QoL in women diagnosed with CC post-diagnosis. The research was part of a larger initiative examining the psychosexual factors influencing QoL in CC patients, focused on physical, psychological, and sexual functioning.

## Materials and methods

This research was conducted at the Oncology Center in Bydgoszcz, Poland, specifically in the Radiotherapy and Clinical Brachytherapy departments. Approval from the Bioethics Committee of the Nicolaus Copernicus University in Toruń functioning at Collegium Medicum in Bydgoszcz and informed consent were acquired. The study was conducted in accordance with all laws and regulations relating to the Declaration of Helsinki.

### Participants

The study involved 60 women diagnosed with stage IIb–IIIa CC before radiotherapy and brachytherapy. The inclusion criteria for participants in the study were as follows: (1) individuals in the age range 40–65, representing middle adulthood; (2) individuals diagnosed with CC in stages II to III based on the International Federation of Gynecology and Obstetrics (FIGO) classification; (3) individuals undergoing either radiotherapy or brachytherapy; (4) individuals who were not admitted for the surgery; and (5) individuals without any medical conditions that could potentially impact their sexual functioning.

### Measures

The 36-Item Short Form Survey (SF-36), which is a component of the Medical Outcomes Study (MOS), was conducted to assess QoL. The SF-36 involves subscales evaluating eight health concepts (physical functioning, role-physical, bodily pain, general health, vitality, social functioning, role-emotional, mental health), organized into two components: physical and mental functioning [15]. The required permissions from the authors of the questionnaire were obtained. The reliability in the study was α = 0.93.

Moreover, the 6-item Female Sexual Function Index (FSFI) was used to indicate the sexual functioning QoL component. The survey measures six domains, including sexual desire, arousal, lubrication, orgasm, satisfaction, and pain [16]. The reliability in the study was α = 0.87.

The Sexual Communication Self-Efficacy Scale (SCSES) was employed to assess the respondents’ confidence in discussing various sexual topics. The 22 items yield five factors: contraception communication, condom negotiation, positive sexual messages, negative sexual messages, and sexual history. The reliability of this scale was 0.98.

In addition, self-administered questions using a “yes–no” scale to assess patient-provider sexual communication were included in the study. These questions focused on discussions about sexual topics with the doctor, initiated by either the healthcare provider or the patient. Specifically, the questions were as follows: (1) Did the doctor discuss potential changes in sexual functioning with you after the cervical cancer diagnosis? (2) If you are experiencing changes in sexual functioning, have you initiated a conversation on this topic with the doctor? These questions were based on previous studies on patient-provider communication in women with gynecological cancer. Following analysis of the results, both questions were consolidated into a single variable: the occurrence of discussions during oncology visits.

Finally, statements from patients about their experiences of sexual communication were included in the study to increase understanding of its role in QoL. Participants in the study gave their consent to be included in the paper. The research was conducted in a face-to-face format, which allowed for the inclusion of patients' personal experiences.

### Statistical analysis

All analyses were performed using IBM’s SPSS (version 26). Descriptive statistics were employed to summarize the demographic and clinical characteristics of the patients. Reliability was assessed using Cronbach’s α. To assess the strength of the relationship between the analyzed variables, the rho-Spearman test and point-biserial correlation were used.

## Results

The sample consisted of 60 women diagnosed with CC. The average age in the CC group was 55.75 (± 6.27) years. Approximately 63.33% of the women resided in urban areas and declared to be in a formal relationship (73.00%). Then, 36.67% (*n* = 22) had vocational education, 30.00% (*n* = 18) had secondary education, and 33.33% (*n* = 20) had higher education. 60.00% (*n* = 36) of the CC patients reported having coexisting medical conditions, most commonly hypertension, thyroid dysfunction, and diabetes. The clinical data of the participants are presented in Table 1. All patients were diagnosed with the II or III stage of CC. They were qualified for treatment with radiotherapy or brachytherapy and had not undergone any previous surgical procedures. The moment of the study was before the initiation of the proper treatment.

**Table 1:** Clinical data related to cervical cancer

	*M*	*SD*
Time between diagnosis and examination (weeks)	4.06	± 1.91
Time between hospital admission and examination (days)	2.13	± 1.23
Treatment type	*n*	*%*
Radiotherapy	29	48.33
Brachytherapy	31	51.67
Disease stage	*n*	*%*
I	0	0.00
II	31	51.67
III	29	48.33
IV	0	0.00

Table 2 refers to the correlations between sexual communication self-efficacy, the occurrence of patient-provider communication, and the quality of life components. It is essential to note that only 14 patients (23.33%) reported discussing their sexual issues with a doctor, initiated either by the healthcare provider or the patient.

**Table 2:** Relations between sexual communication self-efficacy, occurrence of patient-provider communication, and the quality of life components

Variable	Physical functioning QoL component	Psychological functioning QoL component	Sexual functioning QoL component
Sexual history	0.424***	0.520***	0.687***
Condom negotiation	0.394***	0.496***	0.644***
Negative sexual messages	0.445***	0.497***	0.713***
Positive sexual messages	0.443***	0.552***	0.724***
Contraception communication	0.420***	0.536***	0.614***
SCSES general score	0.458***	0.555***	0.713***
Patient-provider sexual communication	0.340**	0.385**	0.333**

*p < 0.05; **p < 0.01; ***p < 0.001

### Sexual communication: personal experiences

Table 3 offers an overview of the participants’ and their partners’ or healthcare providers’ exemplary quotes related to sexual communication.

**Table 3:** Exemplary quotes related to sexual communication between patient and provider

Theme	Example quote
Partner’s sexual communication	1. This sexuality has changed—it’s no longer about sex itself, but about closeness and intimacy. This has also changed; it wasn’t like this before. Now, you could say I take pleasure in that aspect of sex. As if I were about to lose everything, so I take as much as I can
	2. Sex is still important to me—aside from the diagnosis, I’m still a woman and a partner. Especially since a month passed from diagnosis to treatment, I had better and worse moments, so sex didn’t take a back seat, although it wasn’t as frequent. Only the form changes, and I know it’s important for my partner. I won’t escape from it; I still want what’s best for him
	3. There’s one thing, though—this sex is different. I feel worse; I’m always aware that I have cancer. When my husband touches me there, it’s like he’s touching the cancer. That’s why now I gave more, at least I tried
	4. There is no longer closeness between us. I don’t want it; I feel bad when my husband touches my genitals
	5. Sex has taken a back seat; I don't need it anymore; I don’t think about it
	6. I talk a lot with my husband about sex, or perhaps more about intimacy, because sex is not the same anymore, but well, it’s still a part of our life
	7. After all, my husband won't become asexual just because I’m sick; we need to talk. Thanks to discussing sex with my partner, I feel more confident, and we take care of our relationship. I know he won’t leave me, that he’ll be there for me
	8. No, why bother…I have other things on my mind now
Patient-provider sexual communication	9. I don’t talk to the doctor about sex; I’ve never been asked about it. The only thing related to sex is whether I’ve been pregnant
	10. I only talked to the doctor about sex during pregnancy because I had restrictions, but other than that, never
	11. I would like to know how it will be, how my sexuality will change after the illness and treatment because something will definitely change, even just due to the radiotherapy. But I don’t ask because if the doctor doesn’t talk about it, it means it’s not important
	12. The only thing I heard from the doctor is that during treatment, we are prohibited from having intercourse, but he didn’t explain anything more, and I don’t know what happens afterward. I would like to know, but maybe they will tell me about it later
	13. I think it’s a very important topic; sex is a part of our life. I am young, after a divorce, with a new partner, and I would like to know what’s next, but nobody seems to address it. If I don’t ask, I won’t find out. But yes, I talk about it with the doctor; I bring it up myself
	14. Yes, the doctor explained how my sexuality will change and what I can and cannot expect. It reassured me a lot. I told my husband about it; at least, we know what to expect
	15. No, it’s not an important topic. These are not subjects for doctors

## Discussion

Both sexual communication self-efficacy and patient-provider sexual communication were related to all the quality of life components. Additionally, it was proved that the first limitations may appear already at the diagnostic stage, before the initiation of the treatment. Psychosexual changes resulting from the diagnosis, treatment, and side effects of CC indicate that renegotiating one’s own sexual identity and needs is essential [17]. Moreover, it is important to note that the average FSFI-6 scores ranged from 0 to 2.78, with the lowest scores for pain, orgasm, and lubrication and the highest for satisfaction. The pain domain score was zero because no patient reported having had sexual intercourse during the last 30 days, and the questions related only to pain experienced during intercourse. These insights provide a basis for more specific recommendations on key areas to address during consultations. Recent studies have shown the importance of discussions about sexuality in coping with the illness and its side effects, emphasizing that the lack of sexual communication reinforces feelings of inadequacy, isolation, fear, and depression [18].

Following a cancer diagnosis, the importance of closeness and intimacy with partners for women becomes particularly heightened, surpassing pre-diagnosis significance. This generates the necessity to inform the partner about the occurring changes in the body and psyche which explains the relation between sexual communication self-efficacy and sexual functioning. Expressing needs allows for the maintenance of intimacy, contributing to a reduction in uncertainty in the interaction with a partner who is aware of expectations and possibilities [17]. Moreover, effective communication is relevant for partners whose sexual functioning remains unaltered, but whose emotional experiences may give rise to overprotective or avoidance behaviors towards the affected partner [19]. The quotes from participants in this study support the transformative impact of cancer on women’s perceptions of sexuality, shedding light on the complex dynamics within intimate relationships during disease. These narratives underscore the priority for tailored support and open dialogue to address the evolving sexual aspects of life post-diagnosis.

Furthermore, associations between sexual communication and both the physical and psychological QoL components were demonstrated. As sexuality is a significant aspect of QoL and well-being [20], any alterations in this domain may potentially result in a decline in overall QoL [21, 22]. As a unique experience, a cancer diagnosis can diminish the importance of sexual health [23], leading to a decline in sexual functioning and QoL, as along with depressive symptoms and an increased sense of loneliness. Avoiding discussion about sexual topics is known to lead to feelings of helplessness, anger, or depressive symptoms [7], as emphasized in patients’ statements that the period of illness is not conducive to discussing sexual activity. However, effective coping with the changes experienced may also lead to a reduction in the intensity of symptoms related to physical and emotional aspects, as well as improvements in social relationships [22].

In addition, the vast majority of women admitted to never discussing sexual topics with their doctors. For some, it was such an important aspect of functioning, that it led them to inquire about it from healthcare specialists, emphasizing the awareness of these changes happening in their bodies was reassuring to them. Others, despite a need to learn about possible changes in their sexuality, did not raise the issue believing that the doctor should initiate such discussions. There were also statements where patients dismissed sexual topics. Recent studies have confirmed these results, indicating that cancer patients and their relatives want to discuss changes in sexual activity with healthcare professionals but often avoid initiating such discussions, expecting doctors to take the lead [17]. Recent studies indicate that healthcare specialists often avoid sexual topics because of the experienced discomfort, embarrassment, time pressure, lack of appropriate training, shifting the responsibility to patients, or prioritizing other health aspects [24]. It is known that a doctor’s role should also involve challenging the misunderstanding that sexuality in cancer is irrelevant, somewhat permitting couples to discuss sex and intimacy [25]. However, the role of sexual activity is dismissed as the priority is given to somatic changes and consequences of the disease and treatment [26]. Furthermore, in biomedical approach equates sexual activity with penetrative intercourse, which is prohibited during CC treatment [17]. Changing the approach to consider sexual activity as a form of maintaining intimacy and closeness may reduce the marginalization of this issue. Many patients emphasized that sexuality was still important to them. However, there were exceptions among patients who considered sex to be irrelevant in cancer. In such cases, providing information about potential changes and supporting sexual functioning post-treatment may be crucial [17, 27].

In the context of cancer, examining models such as PLISSIT and BETTER is beneficial. The PLISSIT model offers graduated sexual support, starting with “permission” for discussions about sexuality, progressing through “limited information,” “specific suggestions,” and culminating in “intensive therapy” for those requiring specialized help [28]. The BETTER model focuses on five areas: “bringing up,” “explaining” the sexuality needs, “telling resources” to address sexual concerns, “timing” as asking for information anytime, and “Educating” and “Recording” in patients’ medical records [29]. Integrating the PLISSIT and BETTER models can provide a comprehensive view of sexual communication in the context of cancer, enabling the adaptation of support strategies to the individual needs of patients and their partners.

Both models provide a structured approach to sexual health communication. Based on the results of the study, some specific examples can be introduced for the healthcare specialists. Doctors can start by permitting patients to discuss sexual health, thereby normalizing the topic, for example: “It is common for CC treatment to affect your sexual health. Feel free to ask any questions you might have about this.” Then, when the conversion is initiated, healthcare professionals can provide limited, relevant information tailored to the patient’s needs, for example: “Radiotherapy can sometimes cause changes in sexual functions, such as decreased lubrication or discomfort during intercourse.” Based on the patient’s answer, the doctor can offer specific suggestions, like “Using a water-based lubricant can help minimize discomfort.” For patients needing more comprehensive support, it would be proper to refer the patient to a specialist. Combining both models, it would be also relevant to reassure patients that they can ask questions at any time, for example, “You can ask me about this topic at any point in your treatment process.” The study highlights the critical need for healthcare professionals to incorporate sexual communication education into CC care. Improving providers’ communication skills can positively influence various components of patients’ QoL. Proactively addressing psychosexual changes resulting from a CC diagnosis is crucial and requires a patient-centered approach. By challenging misconceptions about sexuality in cancer and emphasizing the role of intimacy, healthcare professionals can contribute to a holistic and supportive care environment, ultimately enhancing the overall well-being of CC patients and their partners. The obtained results indicate also the significance of the above issue already at an earlier stage, after receiving the diagnosis and before starting treatment.

## Conclusions

Overall, this study demonstrates that sexual communication self-efficacy is a significant factor associated with QoL. Moreover, only a few patients in this study discussed sexual issues with their doctors. As patients may experience sexual dysfunction, decreased libido, or changes in preferred forms of intimacy [7], appropriate communication with a partner and doctor about sexual topics will instead allow for the renegotiation of preferences and an improved sexual self-image, while improving QoL.

## Data Availability

Data will be made available by the authors upon reasonable request.
